# Modulation of Autophagy by SARS-CoV-2: A Potential Threat for Cardiovascular System

**DOI:** 10.3389/fphys.2020.611275

**Published:** 2020-11-30

**Authors:** Puneet Kaur Randhawa, Kaylyn Scanlon, Jay Rappaport, Manish K. Gupta

**Affiliations:** ^1^ Division of Metabolic and Cardiovascular Sciences, Burnett School of Biomedical Sciences, College of Medicine, University of Central Florida, Orlando, FL, United States; ^2^ Division of Comparative Pathology, Tulane National Primate Research Center, Covington, LA, United States

**Keywords:** COVID-19, comorbidities, autophagy, heart failure, cytokine storm

## Abstract

Recently, we have witnessed an unprecedented increase in the number of patients suffering from respiratory tract illness caused by severe acute respiratory syndrome coronavirus 2 (SARS-CoV-2). The COVID-19 virus is a single-stranded positive-sense RNA virus with a genome size of ~29.9 kb. It is believed that the viral spike (S) protein attaches to angiotensin converting enzyme 2 cell surface receptors and, eventually, the virus gains access into the host cell with the help of intracellular/extracellular proteases or by the endosomal pathway. Once, the virus enters the host cell, it can either be degraded *via* autophagy or evade autophagic degradation and replicate using the virus encoded RNA dependent RNA polymerase. The virus is highly contagious and can impair the respiratory system of the host causing dyspnea, cough, fever, and tightness in the chest. This disease is also characterized by an abrupt upsurge in the levels of proinflammatory/inflammatory cytokines and chemotactic factors in a process known as cytokine storm. Certain reports have suggested that COVID-19 infection can aggravate cardiovascular complications, in fact, the individuals with underlying co-morbidities are more prone to the disease. In this review, we shall discuss the pathogenesis, clinical manifestations, potential drug candidates, the interaction between virus and autophagy, and the role of coronavirus in exaggerating cardiovascular complications.

## Covid-19 Outbreak as a Pandemic

Severe acute respiratory syndrome coronavirus 2 (SARS-CoV2), a beta coronavirus, is posing a serious threat to humanity all over the globe. Genomic characterization of COVID-19 revealed that the virus might have evolved from bats as the original animal host of the virus and, eventually, infected human beings (Lake, 2020). The unprecedented rise in the infection rate among individuals across the various nations compelled the WHO to regard the disease as a pandemic. As of September 29, 2020, 235 countries are reporting 31,664,104 confirmed cases and 972,221 confirmed deaths, and these numbers only continue to rise. Within the United States alone, there have been a total of 6,874, 982 cases and 200,274 deaths reported by the Centers for Disease Control and Prevention, to date.

Coronaviruses are one of the major groups of viruses, which belong to the Coronaviridae family and can be further subdivided into four genera *viz*. alpha-coronavirus, beta-coronavirus, gamma-coronavirus, and delta-coronavirus based on their serological and phylogenetic clusterization (Schoeman and Fielding, 2019). Coronaviruses have zoonotic origin but six viruses with low pathogenic potential (alpha-coronaviruses: HCoV-229E and HCoV-NL63 and beta-coronaviruses: SARS, MERS-CoV, HCoV-HKU1, and HCoV-OC43) have been found to infect human hosts as well (Fung and Liu, 2019; Guo et al., 2020c). Coronavirus virions are spherical or pleomorphic in shape and their diameters range from 80 to 125 nm (Neuman et al., 2006; Fung and Liu, 2019).

## Genomic Structure and Key Viral Components

**Figure 1:**
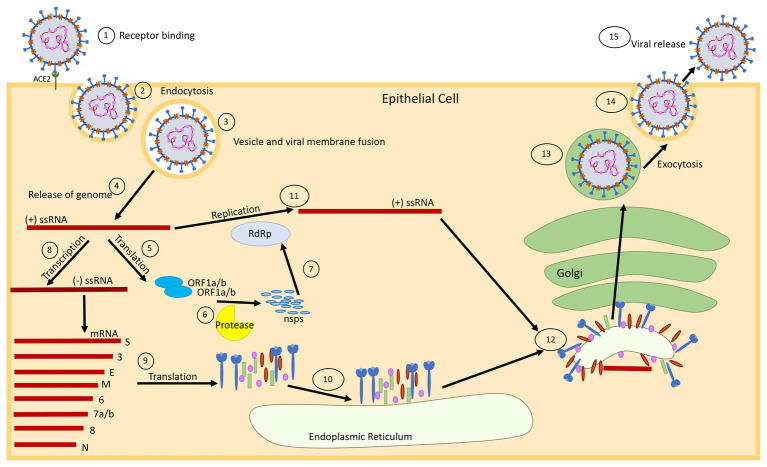
Schematic diagram showing the replication cycle of SARS-CoV-2. (1) SARS-CoV-2 binds to epithelial cells *via* the ACE2 receptor. (2) The virus enters the cell *via* ACE2 mediated endocytosis. (3) The virus membrane and endosome membrane fuse (4) releasing the positive-sense (+) single-stranded RNA (ssRNA) genome into the cytoplasm. (5) The genome is translated to produce ORF1a and ORF1b proteins. (6) These proteins are cleaved into 16 non-structural proteins (nsps) by papain-like protease in a process called proteolysis. (7) The nsps are used to make an RNA-dependent RNA-polymerase (RdRp), which will help with replication of the genome. (8) The original (+) ssRNA genome is also transcribed into negative-sense (−) ssRNA, which is then transcribed into mRNA. (9) The mRNA is translated into the respective structural proteins that (10) gather at the endoplasmic reticulum (ER). (11) The genome is then replicated at full-length, with the help of the RdRp, and assembles with the proteins at the Golgi for packaging. (12) The proteins bud from the ER using the ER membrane (which will become the viral membrane), and migrate to the Golgi apparatus, where the viral package is transported through the Golgi and assembled for (13 and 14) exocytosis. (15) The fully formed virus is then released from the cell surface.

Coronaviruses possess the largest known single-stranded positive-sense RNA genome that ranges from 26 to 32 kb in size (Lu et al., 2020). The genome consists of a 5' cap structure accompanied with a 3' poly-A tail, permitting the virus to act as an mRNA for carrying out the translation of replicase polyproteins (Fung and Liu, 2014). Coronavirus particles comprised four main structural proteins including the spike (S), membrane (M), envelope (E), and nucleocapsid (N; Figure 1; Schoeman and Fielding, 2019; Lu et al., 2020). The N protein binds with the viral RNA and packages the genome into virions. A homotrimeric spike (S) protein protrudes from the surface of the viral envelope (E), which plays a critical role in viral assembly, release, and maintenance of the viral pathogenicity (Fung and Liu, 2019). The M protein is a viral membrane protein, which is known to help the virus in assembly and budding process (Siu et al., 2008). The genome has multiple open reading frames (ORFs) for encoding the accessory proteins along with two large polypeptide coding genes known as ORF1a and ORF1b, which encode 16 non-structural proteins (nsps) to form the coronavirus replicase complex (Figure 1; Chen et al., 2020c).

## SARS-CoV Induces Cellular Stress and Apoptosis

**Figure 2:**
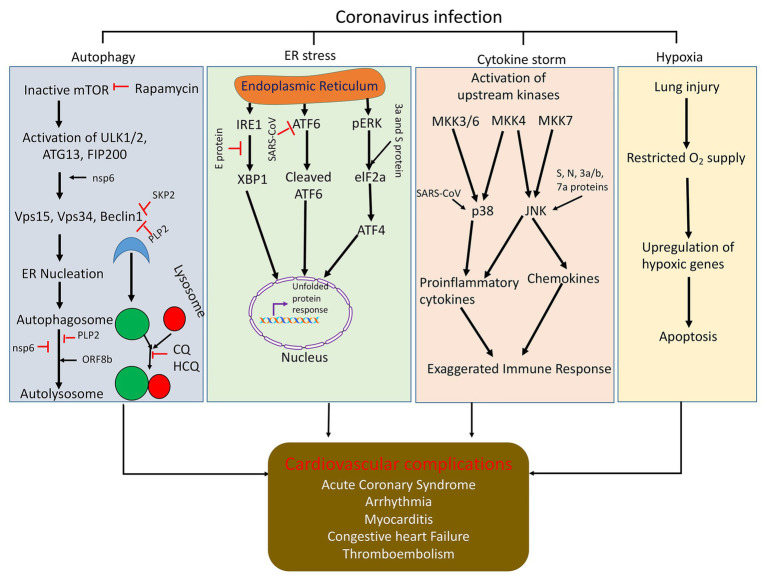
Schematic representation of how coronavirus disrupts signaling pathways (autophagy, ER stress, cytokine storm, and hypoxia) and induces cell death. Autophagy is initiated by the formation of ULK1/2-ATG13-FIP200 complex and then generation of Beclin1 complex and initiation of autophagy vesicle formation. Coronaviral proteins can regulate autophagy through modulating function of autophagy regulatory protein at different stages of autophagy. Viral infection can inhibit ER function and induces ER stress and cell death. Viral protein expresses in ER and upregulates downstream ER stress signaling through modulation the function of ATF6, IRE1 and pERK, and elF2a. Upstream kinases in the JNK and p38 signaling pathways are activated upon infection with SARS-CoV. This activates the mitogen-activated protein kinase kinases (MKK3/6, MKK4, and MKK7). Mkk3/6 and Mkk4 activate p38. SARS-CoV can also directly activate p38 or the SARS-CoV E protein can activate syntenin, which in tune activates p38. These pathways activate proinflammatory cytokine production. MKK4 and MKK7 can also activate JNK. SARS-CoV s, N, 3a, 3b, and 7a proteins can also directly activate JNK. This pathway activates proinflammatory cytokine production and induction of apoptosis. Also, due to viral infection in the lungs, there is restricted supply of O_2_ in the blood, which generate hypoxia and cell death and major organ dysfunction; CQ, chloroquine and HCQ, hydroxychloroquine.

Previous studies have revealed that SARS-CoV has several ORFs including ORF-3a, ORF3b, ORF6, ORF7a, and ORF8 that play critical roles in inducing apoptosis (Law et al., 2005b; Nelson et al., 2005; Yuan et al., 2006; Ye et al., 2008; Shi et al., 2019). ORF3a is exclusively expressed in the ER and Golgi Apparatus and is found to be localized in the nucleolus and the mitochondria. ORF3a is believed to participate in the upregulation of fibrinogen subunits (Aα, Bβ, and γ) and subsequent induction of chromatin condensation, followed by DNA fragmentation in the lung epithelial cells (Law et al., 2005b). ORF3b induces G0/G1 arrest followed by apoptosis after being transfected into the cells. Apparently, ORF6 localizes in the ER and Golgi membrane of infected cells (Chow et al., 2005) and induces apoptosis *via* the caspase-3 dependent pathway and possibly through the phosphorylation of JNK (Figure 2; Ye et al., 2008). However, ORF-7a typically localizes in the ER and has the potential to induce apoptosis *via* activating caspases (Figure 2; Ye et al., 2008). ORF8b is found to form cellular aggregates and induces cell death through ER stress and robust activation of the NLRP3 inflammasome (Shi et al., 2019). Very recently, the ORF8 gene has been considered as a novel target for identifying COVID-19 disease (Kakhki et al., 2020). Besides this, it has been demonstrated that the E protein of SARS-CoV also elicits an immune response to produce apoptosis of the host cell through T-cell mediated immunity, which can be successfully inhibited by Bcl-xL, an anti-apoptotic protein (Yang et al., 2005). Apart from this, the spike protein of SARS-CoV alone was also observed to induce apoptosis *in vitro* (Chow et al., 2005). The SARS-CoV membrane (M) protein has also been implicated to cause apoptosis *via* disruption of the PDK1-PKB/Akt cell survival signaling pathway (Tsoi et al., 2014). These viruses encode pro-apoptotic or anti-apoptotic proteins such that they can either initiate or delay the progression of apoptosis (Ye et al., 2008). The deferment of apoptosis until the late stages of infection impedes the generation of inflammatory response in the host against the virus and facilitates the dissemination of virus into the whole system (Ye et al., 2008). SARS-CoV protein expression can generate protein aggregates *via* inhibiting the cellular protein quality control thereby increasing endoplasmic reticulum stress (ER stress) such that it causes cell death. Induction of ER stress triggers the activation of inositol-requiring enzyme (IRE1), activating transcription factor ATF6, and protein kinase RNA-like ER kinase (PERK). Activation of these enzymes or proteins either brings about ER homeostasis or apoptosis as an antiviral response. Also, the expression of SARS-CoV proteins can have variant effects on numerous proteins to sequester the host’s cell response in order to spread the infection (Fung et al., 2016; Figure 2). This gives us a clear indication that targeting the viral machinery, ranging from ORF genes to spike/envelope protein, may aid in hindering the replication of virus and dissemination of infection in the host.

## Attachment and Entry of Covid-19

Angiotensin converting enzyme 2 (ACE2) is an ectoenzyme expressed in the lungs, heart, kidney, intestine, pancreas, brain, and tongue (Hamming et al., 2004; Guo et al., 2020b), which favors the attachment and entry of the virus into the host cell. In the case of COVID-19 infection, the viral spike (S) protein attaches to the specific cell receptors, for example, ACE2 (found in epithelial cells), which allows the entry of the virus into the cell *via* endocytosis or pore formation (Figure 1; Fung and Liu, 2019). After binding the host ACE2 receptor, the enveloped virus can gain access into the host cells with help of intracellular or extracellular proteases (trypsin, thermolysin, and TMPRSS2), which result in pore formation (Matsuyama et al., 2005; Kim et al., 2020). Interestingly, it has also been reported that SARS-CoV-2 can even enter the cell independently of the cell proteases after being preactivated by proprotein convertase furin (Shang et al., 2020) and the entry can be blocked in the presence of a protease inhibitor (Hoffmann et al., 2020). The cleavage of the spike protein *via* a host protease releases the fusion peptide, which subsequently allows entry into the host cell and eventually the replication process in the host commences (Millet and Whittaker, 2015).

## Clinical Symptoms of Covid-19

In humans, coronaviruses can cause a number of respiratory diseases ranging from the common cold to SARS (Fung and Liu, 2019; Hui and Zumla, 2019). The majority of COVID-19 cases range from asymptomatic to mild but a subset of cases exhibit severe disease. Comorbid conditions such as hypertention, obesity, cardiovascular, and/or pulmonary disorder are known to increase the risk of infection. In severe cases, the transmission of the virus into an individual typically impairs the respiratory system such that it causes difficulty in breathing, cough, fever, tightness in the chest, and dyspnea (Lake, 2020). A more severe form of the illness can produce acute respiratory distress syndrome (ARDS) and pneumonia-like symptoms (Huang et al., 2020a; Lake, 2020). Also, COVID-19 patients display gastrointestinal symptoms including anorexia, nausea, vomiting, diarrhea, and abdominal pain (Pan et al., 2020; Tian et al., 2020). Some infected patients also show symptoms like anosmia, myalgia, muscle soreness, ocular inflammation, headache, dizziness, and altered mental status (Han et al., 2020a; Heidari et al., 2020; Mao et al., 2020; Sun et al., 2020; Wu et al., 2020). Recent studies suggested that SARS-CoV-2 infection shows COVID-19 like symptoms in macaques, which could be used as a model for development of therapeutics (Blair et al., 2020; Rockx et al., 2020).

## Cytokine Release Syndrome

Accumulating evidences have indicated that COVID-19 disease is characterized by an abrupt upsurge in the levels of proinflammatory cytokines, which is known as cytokine storm (Teijaro, 2017; Chen et al., 2020b; Huang et al., 2020a; Ruan et al., 2020a; Zhang et al., 2020). It has been documented that the early infection with SARS CoV-2 is characterized by lymphopenia and is accompanied by the decline in levels of CD4^+^ and CD8^+^ T cells. This causes delay in clearance of the virus but later leads to hyper stimulation of macrophages as well as neutrophils (Wang et al., 2020d). Lymphocytes serve as the determinants for maintaining immune homeostasis and innate immune response (Tan et al., 2020). It has been revealed that the ACE2 receptors are expressed in the lymphocytes, residing in the oral mucosal cavity (Xu et al., 2020a). Therefore, it can be postulated that the presence of virus titers in these lymphocytes may induce infection and even cause death of the lymphocytes. This can be one of the possible contributing factors for the acute decline in the number of lymphocytes. Also, the ability of the virus to directly infect the lymphatic organs cannot be undermined. Recently, Tan et al. reported that the percentage of lymphocytes indicate the disease severity and the chances of recovery (Tan et al., 2020). This suggests that the lymphocyte count can be considered as one of the key indicators of disease progression.

Previous *in-vitro* studies involving infection of macrophages with SARS-CoV indicated that the virus induced the expression of chemotactic proteins, but they produced very low levels of interferons, which are considered as decisive elements in activating an immune response against viral infections (Cheung et al., 2005). In corroboration with the above study, SARS-CoV infection in dendritic cells also led to reduced production of interferons but was associated with a modest increase in the formation of proinflammatory cytokines (TNF-α and IL-6, interleukin-6). However, there was also a remarkable surge in the levels of inflammatory chemokines (macrophage inflammatory protein 1α, regulated on activation normal T cell expressed and secreted, interferon-inducible protein-10, and monocyte chemoattractant protein-1; Law et al., 2005a). This suggests that the viral infection produces a delayed but exaggerated immune response. Studies have also shown that the infection with SARS-CoV can trigger the mitogen-activated protein kinase (MAPK) cascade to induce the release of proinflammatory cytokines through the activation of p38 signaling (Figure 2; Fung et al., 2016).

Intruingly, IL-6 plays a crucial role in the modulation of inflammation (Jones and Jenkins, 2018), and it has been demonstrated to monitor the progression of cytokine release syndrome (Zheng et al., 2020). Furthermore, the level of IL-6 in plasma has been correlated with progression of COVID-19 disease. Several therapeutic approaches are now undergoing clinical trials targeting IL-6 to combat COVID-19 infection (Zhang et al., 2020). As the disease progresses, the COVID-19 patients witness a remarkable increase in the levels of cytokines *viz*. IL-2, IL-7, IL-10, granulocyte-colony stimulating factor, interferon-γ inducible protein 10, monocyte chemoattractant protein 1, macrophage inflammatory protein 1-α, tumor necrosis factor-α (Huang et al., 2020a), and IL-6 (Ruan et al., 2020a; Wang et al., 2020d). This uncontrolled production of proinflammatory cytokines increases the vascular permeability causing unrestricted accumulation of fluid and blood cells into the alveoli. This results in dyspnea and respiratory distress (Leiva-Juarez et al., 2018). Ruan et al. also reported that one of the major causes for COVID-19-associated mortality was virus-induced cytokine storm (Ruan et al., 2020a). In fact, Liu et al. reported that the unrestricted cytokine production in the vascular system can produce diffuse microangiopathy with thrombosis, myocarditis, acute coronary syndrome, arrhythmia, and even multi-organ failure (Wang et al., 2020d). Consistent with the human data, in a recent experiment with nonhuman primates, it was also discovered that African green monkeys infected with SARS-Cov-2 show strong cytokine storm signal along with ARDS symptoms (Blair et al., 2020). This suggests that limiting the release of cytokines may restrict the development and progression of inflammation in various organs including the heart.

## Cardiovascular Disorder in Covid-19 Patients

It has been reported that the patients with pre-existing cardiovascular disease are at increased risk of developing cardiac dysfunctioning as well as heart failure upon being infected (Driggin et al., 2020; Group et al., 2020). A study from Wuhan, China (where the disease was first identified) reported that out of 44,672 confirmed cases, 10.5% of patients had cardiovascular disorder (CVD) and 6.0% had hypertension. COVID-19 patients with comorbidities also have a higher case-fatality rate as well (Driggin et al., 2020). Moreover, a meta-analysis also revealed that COVID-19 infection may exaggerate myocardial damage in patients with pre-existing cardiovascular diseases (Group et al., 2020). Analysis of autopsy samples from COVID-19 patients suggests that the viral infection may cause cardiomegaly, right ventricular dilation, and generation of scattered myocytes necrosis, which may compromise heart function (Fox et al., 2020). Furthermore, a significant fraction of COVID-19 patients develop arrhythmias, which suggest that COVID-19 patients have compromised heart function (Driggin et al., 2020). Troponin I, a known marker of cardiovascular injury (Collins et al., 2001), was found to be significantly increased in COVID-19 patients, especially with severe disease (Lippi et al., 2020). Also, various authors have reported an upsurge in the level of creatine kinase isoenzyme-MB (CK-MB), myohemoglobin (MYO), lactate dehydrogenase, and N-terminal pro-brain natriuretic peptide after COVID-19 infection (Han et al., 2020b; Wang et al., 2020b; Zeng et al., 2020). In fact, some studies have reported circulatory heart failure in some patients (Ruan et al., 2020b; Table 1).

**Table 1:** Cardiovascular complications caused by SARS-CoV-2 virus.

S. No	Clinical presentation	Total participants (N)	Median age	Markers of injury	References
1	Acute cardiac injury	416	64	Cardiac Troponin I	Shi et al., 2020
2	Acute cardiac injury	273	58.5	Creatine kinase isoenzyme-MB (CK-MB) Myohemoglobin (MYO) Cardiac troponin I N-terminal pro-brain natriuretic peptide	Han et al., 2020b
3	Coronary heart disease	150	N/A	N-terminal pro B-type natriuretic peptide Cardiac troponin-I	Chen et al., 2020a
4	Arrhythmia	138	56	Lactate dehydrogenase	Wang et al., 2020b
5	Circulatory failure	68	N/A	Myoglobin, Cardiac troponin	Ruan et al., 2020b
6	Acute cardiac injury	269	60	Cardiac troponin I	Li et al., 2020
7	Malignant arrhythmia	187	58.5	Cardiac troponin T N-terminal pro-brain natriuretic peptide	Guo et al., 2020a
8	Myocarditis and heart failure	1	63	Myohemoglobin (MYO) Cardiac troponin I N-terminal pro-brain natriuretic peptide	Zeng et al., 2020

Thus, COVID-19 infection can aggravate cardiovascular complications in patients with cardiovascular perturbations. A study by Hanley et al. suggested that COVID-19 patients have signs of active viral infection in the cardiac tissue along with pericarditis and necropsy of endocardial tissue, which may lead to cardiac hypertrophy and functional abnormalities (Hanley et al., 2020). Since ACE2 is implicated in monitoring heart function and hypertension development, the high expression of ACE2 both in heart and lung epithelial tissue can be a cause of increased myocardial injury through increased secretion of ACE2 (Zheng et al., 2020). Though it is known that SARS-CoV-2 can infect epithelial cells, discovering the mechanism of infection causing heart complications is still under investigation.

## Interplay Between Autophagy and Coronavirus

The interaction between the virus and the host cell is one of the major predetermining factor influencing autophagy-mediated responses in order to combat viral infection. The implication of autophagy in monitoring the progression of viral infection has been investigated by various scientists (Reggiori et al., 2010; Gassen et al., 2019). Autophagy is a quintessential cellular process that is responsible for degrading the damaged cytosolic proteins, intracellular pathogens, and dysfunctional organelles through a double-membrane organelle referred to as autophagosome (Levine and Kroemer, 2019; Yang and Shen, 2020). The autophagosome, eventually fuses with the lysosome, which results in the formation of the autolysosome, the components of which are then degraded by lysosomal enzymes (Levine and Kroemer, 2019; Yang and Shen, 2020). It is manifested that the cell can modulate the autophagy level to maintain homeostasis and counter infection in the cell. Degradation of virus *via* the autophagy process can provide inbuilt defense against the infection (Cottam et al., 2011). Autophagy can either be non-selective (induced by starvation or other stressor stimuli) or selective, whereby the accumulation of specific adaptor proteins including p62, BCL2 and adenovirus E1B 19-kDa-interacting protein 3 (BNIP3), NIP3-like protein X (NIX), and neighbor of BRCA1 gene1 (NBR1) initiate selective autophagy *via* recognizing ubiquitinated protein organelles or complexes and ultimately form autophagosome for degradation (Chiramel et al., 2013; Nascimbeni et al., 2017). The pattern recognition receptors *viz*. toll like receptors recognize pathogen associated molecular patterns (PAMPs) and trigger autophagy and the synthesis of inflammatory cytokines and interferons to generate an anti-viral response (Choi et al., 2018).

The induction of autophagy is a coordinated maneuver involving active participation class III phosphatidylinositol 3-phosphate (PtdIns 3 P), Unc-51 like autophagy activating kinase (ULK1), and autophagy related (Atg-16L1) protein complex at different stages of autophagosome formation. ULK1 complex comprises ULK1, ATG13, RB1CC1, and ATG101; PtdIns 3 P comprises ATG14, BECN1, PIK3R4, and PIK3C3; and ATG16L1 complex comprises ATG16L1, ATG5, and ATG12 (Bello-Perez et al., 2020). Mammalian target of rapamycin (MTOR) is the prime switch to control the initiation of autophagy as it phosphorylates and inactivates ULK complex under resting conditions (Bello-Perez et al., 2020). However, under starving or stressful conditions MTOR is inactivated and the ULK complex is activated, which in turn activates PtdIns 3 P complex to promote the formation of PtdIns 3 P regions on the periphery of a specific structure. PtdIns 3 P binds to FYVE-domain containing protein Zinc finger FYVE domain-containing protein 1 (ZFYVE1) to give rise to specific structure called omegasome, which is regarded as a matrix for autophagosome biogenesis (Nascimbeni et al., 2017). The non-structural protein (Nsp6) can induce these domains and facilitate recruitment of PtdIns 3 P effector proteins to form an autophagosome (Cottam et al., 2014; Bello-Perez et al., 2020; Carmona-Gutierrez et al., 2020). However, it has been reported that Nsp6 dependent autophagy induction produces significantly small diameter autophagosomes in comparison to the ones induced by starvation or other stressors. This possibly limits the expansion of autophagosomes, restricts their capacity to fuse with numerous lysosomes to generate big autolysosome and in turn increase the number of autophagic vesicles at early stage of autophagy (Cottam et al., 2014). The omegasome gives rise to phagophore (pre-autophagosomal double membrane structure), which upon elongation and sealing forms mature autophagosome. The phagophore elongation depends on PtdIns 3 P-WIPI2 (WD repeat domain phosphoinositide-interacting protein 2) interaction. WIPI2 regulates the assembly of Atg conjugation system and facilitates insertion of cytosolic LC3II into the autophagosomes. The level of autophagy is determined either by the intracellular level of LC3II or the movement of cytosolic LC3I into LC3II positive double membrane vesicles. Conventionally, LC3 was regarded as a marker of autophagosome formation in mammalian cells but any of the Atg8 family member can serve as autophagosome marker (Chiramel et al., 2013; Nascimbeni et al., 2017). Apparently, the viral infection can augment autophagosome abundance either *via* generating immature autophagosomes or mitigating their degradation (Chiramel et al., 2013; Nascimbeni et al., 2017; Carmona-Gutierrez et al., 2020). Thus, owing to the dynamic nature of autophagy, it is necessary to evaluate the autophagic flux to comprehend the actual manner, the viral infection affects autophagy in the cells.

As described earlier, the core autophagic machinery, being an indispensable part of immune system, senses the presence of virus and mounts an anti-viral defense response. However, some viruses subvert the autophagic response and exit the autophagic process without lysis or block autophagic degradation at the final stage (Chiramel et al., 2013; Granato et al., 2014; Jackson, 2014). It has been reported that herpes simplex virus encodes a neurovirulence factor ICP34.5, which can counter regulate the activity of eukaryotic initiation factor-2-α (eIF2α), beclin, and TANK-binding kinase to block autophagosome maturation (Talloczy et al., 2002; Kanai et al., 2012; Choi et al., 2018). In corroboration with the above study, Chaumorcel et al. reported that human cytomegalovirus stimulates autophagy at the early stages of infection (increases autophagic flux) but blocks autophagy at later stages of infection *via* mutual interaction between a virulence factor TRS1 and beclin protein (Chaumorcel et al., 2012; Choi et al., 2018). Also, Kaposi’s sarcoma associated herpes virus encodes a functional homolog of B-cell lymphoma 2 (Bcl-2), which mimics cellular Bcl-2 and exhibits the potential to mitigate autophagy *via* interacting with Beclin protein (Cuconati and White, 2002; Choi et al., 2018). Rubicon operates as a part of a Beclin-1-Vps34 autophagy complex (Kim et al., 2017), and it has been reported that K7 protein of Kaposi’s sarcoma associated herpes virus facilitates Beclin-Rubicon interaction and prohibits Vps34 (class III phosphatidylinositol-3 kinase) activity to prevent the fusion of autophagosome with lysosome (Liang et al., 2013). Besides this, Kaposi’s sarcoma associated herpes virus Flice-like inhibitory protein (FLIP) homolog can inhibit Atg3 to bind and process LC3 during the course of autophagosome elongation to limit autophagy. Also, during the early course of human immunodeficiency virus infection, there is an increased autophagosome formation and a HIV-1 Gag (structural protein) colocalized with endogenous LC3. However, HIV1 negative factor (Nef; prerequisite for replication of virus) interacts with Beclin to prohibit the maturation of autophagosomes (Kyei et al., 2009).

Apparently, the blockade of autophagy by different types of viruses gives us an indication that viruses can manipulate autophagy for immune evasion. The notion that coronavirus requires the formation of double membrane vesicles to aid replication and transcription of the virus gives us an indication that the virus may usurp the autophagosomal machinery to facilitate the formation of double membrane vesicles (Carmona-Gutierrez et al., 2020). Thus, autophagosomes boost infection by assisting the assembly of viral replicase proteins (Cottam et al., 2011). This is further corroborated by the fact that viral nsp6 protein was found to colocalize with the endogenous LC3, indicating a possible co-relation between autophagy and coronavirus replication (Cottam et al., 2011; Bello-Perez et al., 2020). Also, it has been reported that coronaviruses can hijack the EDEMosome (vesicles for endoplasmic reticulum degradation) formation pathway *via* modulating the degradation of endoplasmic reticulum degradation enhancing alpha-mannosidase like protein 1 (EDEM1) and OS-9 protein such that it causes accumulation of both the endoplasmic reticulum-associated degradation (ERAD) regulatory proteins and trap them into double-membrane vesicles (Reggiori et al., 2010). This indicates that if the virus evades autophagy, it can dynamically manipulate autophagy to promote the replication of the virus inside the host.

Various scientists have revealed that Beclin 1 can serve as a potential target to restrict the multiplication of the virus in the host (Gassen et al., 2019). Beclin 1 fairly regulates the autophagy pathway to restrict the multiplication of the virus inside the host cell (Kang et al., 2011; Gassen et al., 2019). Gassen et al. reported that S-phase kinase-associated protein 2 (SKP2), an E3 ligase, is responsible for carrying out poly-ubiquitination and subsequent proteasomal degradation of Beclin 1 (Figure 2). The authors witnessed a significant decline in the levels of Beclin 1 protein and successive fusion of autophagosomes with the lysosomes as MERS-CoV started replicating inside the cell. However, inhibition of SKP2 reduced Beclin 1 ubiquitination, consequent degradation, and also enhanced the autophagic flux. The authors reported that SKP2 inhibition enhanced autophagy and also ameliorated the replication of MERS-CoV (Gassen et al., 2019). Thus, the authors proposed that SKP2-Beclin 1 can serve as a potential target for antiviral drugs to reduce the virus multiplication. SARS-CoV can also sequester the autophagy pathway to inhibit the formation of the autolysosome *via* the nsp6 of SARS-CoV (Figure 2). Although nsp6 protein activates autophagy, the autophagy flux becomes dysregulated (as mentioned earlier) to favor viral replication (Fung and Liu, 2019). Another *in vitro* study revealed that the membrane-associated papain-like protease 2 (PLP2) of coronaviruses induces inadequate autophagy and commences replication by increasing the buildup of autophagosomes *via* prevention of the autophagosomal-lysosomal fusion (Chen et al., 2014). On the contrary, Zhao et al. reported that an intact autophagic pathway is not a prerequisite for spreading the viral infection in the host (Zhao et al., 2007).

Based on the ability to modulate the autophagic pathway, various unproven drugs like chloroquine, hydroxychloroquine, azithromycin, or their combinations were administered to the infected patients to combat SARS-CoV-2 infection (Gao et al., 2020). The notion that these drugs could prevent the endocytic pathway, which subsequently prevents the virus replication, gave a compelling indication to incorporate these medications in the drug regimen of the infected masses (Gao et al., 2020). It was found that hydroxychloroquine reduced the mortality rate in critically ill patients suffering from COVID-19 (Meo et al., 2020; Yu et al., 2020), but some studies indicated that these autophagy modulators failed to ameliorate the viral load in the infected individuals (Molina et al., 2020; Singh et al., 2020) and lead to prolongation of QT interval (Chorin et al., 2020; Jankelson et al., 2020). This indicates that further studies need to be envisaged to validate the usage of autophagy modulators in order to deter the progression of infection.

## Potential Drugs for the Treatment of Covid-19

There are a few anti-viral drugs that are being tested for their potential to attenuate COVID-19 viral infection (Parang et al., 2020; Wang et al., 2020c,d). These include remdesivir, lopinavir, ritonavir, chloroquine, and hydroxychloroquine. Remdesivir is a prodrug (adenosine nucleotide analog) and its metabolite inhibits viral RNA polymerases to elicit anti-viral action. Remdesivir has been recognized as an antiviral drug against a wide range of RNA viruses including bat coronavirus, SARS-CoV, MERS-CoV, and human coronavirus 229E (Sheahan et al., 2017; Parang et al., 2020). Sheahan et al. showed that remdesivir administration significantly abrogated SARS-CoV and MERS-CoV replication in primary human airway epithelial cell cultures (Sheahan et al., 2020). Furthermore, remdesivir administration in mice also decreased lung viral load and improved respiratory function (Sheahan et al., 2020). Recently, another *in vitro* study has revealed that remdesivir exhibits anti-viral activity against COVID-19 (Wang et al., 2020c). Efficacy of remdesivir in the treatment of COVID-19 infected patients is under clinical trial and has been approved by FDA to use as an emergency drug. Chloroquine has been conventionally used as an anti-malarial and immunomodulator drug but it also exhibits antiviral potential against coronaviruses (Savarino et al., 2006) and provides shielding effect against viral infection by increasing the pH of endosomes to prevent the fusion of virus and endosomes. Apart from this, in the presence of chloroquine, ACE2 receptors are under-glycosylated and have lesser affinity for coronavirus spike protein (Vincent et al., 2005). Mauthe et al. reported that the chloroquine treatment decreases autophagic flux because of the lack of fusion between autophagosome and lysosome (Mauthe et al., 2018). Liu et al. also reported that administration of chloroquine and hydroxyl-choloroquine subverted the transport of COVID-19 virus from early endosomes to endolysosomes which is pre-requisite for the release of viral genome (Wang et al., 2020d). Apart from this, it has also been reported that chloroquine is a potent vasodilator and can mitigate hypoxia-induced pulmonary hypertension possibly resulting in reduced injury in tissues including heart (Wu et al., 2017).

However, recently, there have been reports that the anti-viral drugs more effectively reduce the viral load in comparison to hydroxychloroquine (Geleris et al., 2020; Musarrat et al., 2020; Nutho et al., 2020; Singh et al., 2020). Musarrat et al. reported that nelfinavir possesses the potential to inhibit the spike glycoprotein dependent fusion of the viral envelope and the plasma/endosomal membrane to prevent the spread of infection (Musarrat et al., 2020). Although there have been reports that hydroxychloroquine reduces the mortality rate in critically ill patients suffering from COVID-19 (Yu et al., 2020), a meta-analysis revealed that this drug does not aid in clearing the viral load and significantly increases the fatality among the infected individuals (Singh et al., 2020). There have also been compelling evidences indicating that chloroquine, hydroxychloroquine, and azithromycin prolong the QT-interval, which may lead to precipitation of arrhythmia (Chorin et al., 2020; Jankelson et al., 2020). This suggests that the benefits of using chlorquine do not overweigh the risks upon using this unproven therapy. Recent study by the Group et al. suggested that use of dexamethasone can shorten the hospitalization time of COVID-19 positive patients along with lower rate of mortality at 28 days of post COVID-19 infection. Dexamethasone showed protective effect in patients who are on additional life supportion devices (Group et al., 2020). As drug/vaccine development and evaluation is a lengthy process, more studies need to be envisaged using existing anti-viral or autophagy modulators to elucidate the possible mechanisms involved in the progression of the disease to confront the SARS-CoV-2 pandemic (Table 2).

**Table 2:** Potential treatment strategies used against SARS-CoV-2 virus.

Treatment strategy	Category	Mechanism of action	References
Remdesivir	Anti-viral (adenosine nucleotide analog)	Decreases RNA replication by reducing RNA dependent RNA polymerase	Parang et al., 2020; Wang et al., 2020c,d
Lopinavir	Anti-viral (protease inhibitor)	Counter regulates 3CLpro, which cleaves the large replicase polyproteins during viral replication	Lim et al., 2020; Liu et al., 2020
Ritonavir	Anti-viral (protease inhibitor)	Counter regulates 3CLpro, which cleaves the large replicase polyproteins during viral replication	Lim et al., 2020; Ye et al., 2020b
Chloroquine	Antimalarial and Autophagy inhibitor	Abrogates endocytic pathways to prevent replication of the virus	Huang et al., 2020b
Hydroxychloroquine	Antimalarial and Autophagy inhibitor	Abrogates endocytic pathways to prevent replication of the virus	Gautret et al., 2020
Azithromycin	Macrolide antibiotic	Prohibits internalization of the virus into the host cell	Tran et al., 2019
Dexamethasone	Anti-inflammatory and immunosuppressant	Reduces the activation of immune system and subsequent production of inflammatory cytokines	Group et al., 2020; Tomazini et al., 2020
47D11 mAb	Monoclonal antibody	Prohibits angiotensin converting enzyme 2 (ACE2)-virus interaction and inhibit the entry of virus.	Wang et al., 2020a
Tocilizumab	Monoclonal antibody	Reduces the level of inflammatory protein IL-6	Melody et al., 2020; Sciascia et al., 2020; Xu et al., 2020b
Lenzilumab	Monoclonal antibody	Targets colony stimulating factor 2/granulocyte-macrophage colony stimulating factor to reduce the systemic inflammatory response	Melody et al., 2020; Temesgen et al., 2020
Telmisartan	Angiotensin receptor antagonist	Mitigates the binding of circulating Angiotensin II to Angiotensin I receptor to reduce vasoconstriction	Rothlin et al., 2020
Convalescent plasma transfusion	Passive immunotherapy	Neutralizes the virus particles	Ye et al., 2020a

## Limitations

Anti-oxidant/novel compounds, which have the capability to modulate autophagy, can be the potential anti-viral candidates and their role in combating anti-viral infection still needs to be explored. Also, the mechanisms and the virulence factor using which the SARS-CoV-2 escapes autophagy need to be explored exclusively.

## Author Contributions

All authors listed have made a substantial, direct and intellectual contribution to the work and approved it for publication.

### Conflict of Interest

The authors declare that the research was conducted in the absence of any commercial or financial relationships that could be construed as a potential conflict of interest.

## References

[ref1] Bello-Perez M. Sola I. Novoa B. Klionsky D. J. Falco A. (2020). Canonical and noncanonical autophagy as potential targets for COVID-19. Cell 9:1619. 10.3390/cells9071619, PMID: 32635598 PMC7408018

[ref2] Blair R. V. Vaccari M. Doyle-Meyers L. A. Roy C. J. Russell-Lodrigue K. Fahlberg M. (2020). ARDS and Cytokine Storm in SARS-CoV-2 Infected Caribbean Vervets. bioRxiv [Preprint]. 10.1101/2020.06.18.157933

[ref3] Carmona-Gutierrez D. Bauer M. A. Zimmermann A. Kainz K. Hofer S. J. Kroemer G.. (2020). Digesting the crisis: autophagy and coronaviruses. Microb. Cell 7, 119–128. 10.15698/mic2020.05.715, PMID: 32391393 PMC7199282

[ref4] Chaumorcel M. Lussignol M. Mouna L. Cavignac Y. Fahie K. Cotte-Laffitte J.. (2012). The human cytomegalovirus protein TRS1 inhibits autophagy via its interaction with Beclin 1. J. Virol. 86, 2571–2584. 10.1128/JVI.05746-11, PMID: 22205736 PMC3302257

[ref5] Chen C. Chen C. Yan J. T. Zhou N. Zhao J. P. Wang D. W. (2020a). Analysis of myocardial injury in patients with COVID-19 and association between concomitant cardiovascular diseases and severity of COVID-19. Zhonghua Xin Xue Guan Bing Za Zhi 48, 567–571. 10.3760/cma.j.cn112148-20200225-00123, PMID: 32141280

[ref6] Chen Y. Liu Q. Guo D. (2020c). Emerging coronaviruses: genome structure, replication, and pathogenesis. J. Med. Virol. 92, 418–423. 10.1002/jmv.25681, PMID: 31967327 PMC7167049

[ref7] Chen X. Wang K. Xing Y. Tu J. Yang X. Zhao Q.. (2014). Coronavirus membrane-associated papain-like proteases induce autophagy through interacting with Beclin1 to negatively regulate antiviral innate immunity. Protein Cell 5, 912–927. 10.1007/s13238-014-0104-6, PMID: 25311841 PMC4259884

[ref8] Chen C. Zhang X. R. Ju Z. Y. He W. F. (2020b). Advances in the research of cytokine storm mechanism induced by corona virus disease 2019 and the corresponding immunotherapies. Zhonghua Shao Shang Za Zhi 36:E005. 10.3760/cma.j.cn501120-20200224-00088, PMID: 32114747

[ref9] Cheung C. Y. Poon L. L. Ng I. H. Luk W. Sia S. F. Wu M. H.. (2005). Cytokine responses in severe acute respiratory syndrome coronavirus-infected macrophages in vitro: possible relevance to pathogenesis. J. Virol. 79, 7819–7826. 10.1128/JVI.79.12.7819-7826.2005, PMID: 15919935 PMC1143636

[ref10] Chiramel A. I. Brady N. R. Bartenschlager R. (2013). Divergent roles of autophagy in virus infection. Cell 2, 83–104. 10.3390/cells2010083, PMID: 24709646 PMC3972664

[ref11] Choi Y. Bowman J. W. Jung J. U. (2018). Autophagy during viral infection - a double-edged sword. Nat. Rev. Microbiol. 16, 341–354. 10.1038/s41579-018-0003-6, PMID: 29556036 PMC6907743

[ref12] Chorin E. Wadhwani L. Magnani S. Dai M. Shulman E. Nadeau-Routhier C.. (2020). QT interval prolongation and torsade de pointes in patients with COVID-19 treated with hydroxychloroquine/azithromycin. *Heart Rhythm* 17, 1425–1433. 10.1016/j.hrthm.2020.05.014, PMID: 32407884 PMC7214283

[ref13] Chow K. Y. Yeung Y. S. Hon C. C. Zeng F. Law K. M. Leung F. C. (2005). Adenovirus-mediated expression of the C-terminal domain of SARS-CoV spike protein is sufficient to induce apoptosis in vero E6 cells. FEBS Lett. 579, 6699–6704. 10.1016/j.febslet.2005.10.065, PMID: 16310778 PMC7094440

[ref14] Collins J. N. Cole F. J. Weireter L. J. Riblet J. L. Britt L. D. (2001). The usefulness of serum troponin levels in evaluating cardiac injury. Am. Surg. 67, 821–825. PMID: 11565757

[ref15] Cottam E. M. Maier H. J. Manifava M. Vaux L. C. Chandra-Schoenfelder P. Gerner W.. (2011). Coronavirus nsp6 proteins generate autophagosomes from the endoplasmic reticulum via an omegasome intermediate. Autophagy 7, 1335–1347. 10.4161/auto.7.11.16642, PMID: 21799305 PMC3242798

[ref16] Cottam E. M. Whelband M. C. Wileman T. (2014). Coronavirus NSP6 restricts autophagosome expansion. Autophagy 10, 1426–1441. 10.4161/auto.29309, PMID: 24991833 PMC4203519

[ref17] Cuconati A. White E. (2002). Viral homologs of BCL-2: role of apoptosis in the regulation of virus infection. Genes Dev. 16, 2465–2478. 10.1101/gad.1012702, PMID: 12368257

[ref18] Driggin E. Madhavan M. V. Bikdeli B. Chuich T. Laracy J. Bondi-Zoccai G.. (2020). Cardiovascular considerations for patients, health care workers, and health systems during the coronavirus disease 2019 (COVID-19) pandemic. J. Am. Coll. Cardiol. 75, 2352–2371. 10.1016/j.jacc.2020.03.031, PMID: 32201335 PMC7198856

[ref19] Fox S. E. Akmatbekov A. Harbert J. L. Li G. Brown J. Q. Vander Heide R. S. (2020). Pulmonary and Cardiac Pathology in Covid-19 The First Autopsy Series from New Orleans. medRxiv [Preprint]. 10.1101/2020.04.06.20050575 PMC7255143 32473124

[ref20] Fung T. S. Liao Y. Liu D. X. (2016). Regulation of stress responses and translational control by coronavirus. Viruses 8:184. 10.3390/v8070184, PMID: 27384577 PMC4974519

[ref21] Fung T. S. Liu D. X. (2014). Coronavirus infection, ER stress, apoptosis and innate immunity. Front. Microbiol. 5:296. 10.3389/fmicb.2014.00296, PMID: 24987391 PMC4060729

[ref22] Fung T. S. Liu D. X. (2019). Human coronavirus: host-pathogen interaction. Annu. Rev. Microbiol. 73, 529–557. 10.1146/annurev-micro-020518-115759, PMID: 31226023

[ref23] Gao J. Tian Z. Yang X. (2020). Breakthrough: Chloroquine phosphate has shown apparent efficacy in treatment of COVID-19 associated pneumonia in clinical studies. Biosci. Trends 14, 72–73. 10.5582/bst.2020.01047, PMID: 32074550

[ref24] Gassen N. C. Niemeyer D. Muth D. Corman V. M. Martinelli S. Gassen A.. (2019). SKP2 attenuates autophagy through Beclin1-ubiquitination and its inhibition reduces MERS-coronavirus infection. Nat. Commun. 10:5770. 10.1038/s41467-019-13659-4, PMID: 31852899 PMC6920372

[ref25] Gautret P. Lagier J. C. Parola P. Hoang V. T. Meddeb L. Mailhe M.. (2020). Hydroxychloroquine and azithromycin as a treatment of COVID-19: results of an open-label non-randomized clinical trial. Int. J. Antimicrob. Agents 56:105949. 10.1016/j.ijantimicag.2020.105949, PMID: 32205204 PMC7102549

[ref26] Geleris J. Sun Y. Platt J. Zucker J. Baldwin M. Hripcsak G.. (2020). Observational study of Hydroxychloroquine in hospitalized patients with Covid-19. N. Engl. J. Med. 382, 2411–2418. 10.1056/NEJMoa2012410, PMID: 32379955 PMC7224609

[ref27] Granato M. Santarelli R. Farina A. Gonnella R. Lotti L. V. Faggioni A.. (2014). Epstein-barr virus blocks the autophagic flux and appropriates the autophagic machinery to enhance viral replication. J. Virol. 88, 12715–12726. 10.1128/JVI.02199-14, PMID: 25142602 PMC4248894

[ref28] Group R. C. Horby P. Lim W. S. Emberson J. R. Mafham M. Bell J. L.. (2020). Dexamethasone in hospitalized patients with Covid-19- preliminary report. N. Engl. J. Med. 10.1056/NEJMoa2021436, PMID: [Epub ahead of print] 32678530 PMC7383595

[ref29] Guo Y. R. Cao Q. D. Hong Z. S. Tan Y. Y. Chen S. D. Jin H. J.. (2020c). The origin, transmission and clinical therapies on coronavirus disease 2019 (COVID-19) outbreak - an update on the status. Mil. Med. Res. 7:11. 10.1186/s40779-020-00240-0, PMID: 32169119 PMC7068984

[ref30] Guo T. Fan Y. Chen M. Wu X. Zhang L. He T.. (2020a). Cardiovascular implications of fatal outcomes of patients with coronavirus disease 2019 (COVID-19). JAMA Cardiol. 5, 811–818. 10.1001/jamacardio.2020.1017, PMID: 32219356 PMC7101506

[ref31] Guo W. Li M. Dong Y. Zhou H. Zhang Z. Tian C.. (2020b). Diabetes is a risk factor for the progression and prognosis of COVID-19. Diabetes Metab. Res. Rev. 36:e3319. 10.1002/dmrr.3319, PMID: 32233013 PMC7228407

[ref32] Hamming I. Timens W. Bulthuis M. L. Lely A. T. Navis G. van Goor H. (2004). Tissue distribution of ACE2 protein, the functional receptor for SARS coronavirus. A first step in understanding SARS pathogenesis. J. Pathol. 203, 631–637. 10.1002/path.1570, PMID: 15141377 PMC7167720

[ref33] Han C. Duan C. Zhang S. Spiegel B. Shi H. Wang W.. (2020a). Digestive symptoms in COVID-19 patients with mild disease severity: clinical presentation, stool viral RNA testing, and outcomes. Am. J. Gastroenterol. 115, 916–923. 10.14309/ajg.0000000000000664, PMID: 32301761 PMC7172493

[ref34] Han H. Xie L. Liu R. Yang J. Liu F. Wu K.. (2020b). Analysis of heart injury laboratory parameters in 273 COVID-19 patients in one hospital in Wuhan, China. J. Med. Virol. 92, 819–823. 10.1002/jmv.25809, PMID: 32232979 PMC7228305

[ref35] Hanley B. Naresh K. N. Roufosse C. Nicholson A. G. Weir J. Cooke G. S. (2020). Histopathological findings and viral tropism in UK patients with severe fatal COVID-19: a post-mortem study. Lancet Microbe. 1, e245–e253. 10.1016/S2666-5247(20)30115-4 32844161 PMC7440861

[ref36] Heidari F. Karimi E. Firouzifar M. Khamushian P. Ansari R. Mohammadi Ardehali M.. (2020). Anosmia as a prominent symptom of COVID-19 infection. Rhinology 58, 302–303. 10.4193/Rhin20.140, PMID: 32319971

[ref37] Hoffmann M. Kleine-Weber H. Schroeder S. Kruger N. Herrler T. Erichsen S.. (2020). SARS-CoV-2 cell entry depends on ACE2 and TMPRSS2 and is blocked by a clinically proven protease inhibitor. Cell 181, 271–280.e8. 10.1016/j.cell.2020.02.052, PMID: 32142651 PMC7102627

[ref38] Huang M. Tang T. Pang P. Li M. Ma R. Lu J.. (2020b). Treating COVID-19 with Chloroquine. J. Mol. Cell Biol. 12, 322–325. 10.1093/jmcb/mjaa014, PMID: 32236562 PMC7232130

[ref39] Huang C. Wang Y. Li X. Ren L. Zhao J. Hu Y. (2020a). Clinical features of patients infected with 2019 novel coronavirus in Wuhan, China. Lancet 395, 497–506. 10.1016/S0140-6736(20)30183-5 31986264 PMC7159299

[ref40] Hui D. S. C. Zumla A. (2019). Severe acute respiratory syndrome: historical, epidemiologic, and clinical features. Infect. Dis. Clin. N. Am. 33, 869–889. 10.1016/j.idc.2019.07.001, PMID: 31668196 PMC7127569

[ref41] Jackson W. T. (2014). Dangerous membranes: viruses that subvert Autophagosomes. EBioMedicine 1, 97–98. 10.1016/j.ebiom.2014.11.015, PMID: 26137514 PMC4457410

[ref42] Jankelson L. Karam G. Becker M. L. Chinitz L. A. Tsai M. C. (2020). QT prolongation, torsades de pointes, and sudden death with short courses of chloroquine or hydroxychloroquine as used in COVID-19: A systematic review. *Heart Rhythm* 17, 1472–1479. 10.1016/j.hrthm.2020.05.008, PMID: 32438018 PMC7211688

[ref43] Jones S. A. Jenkins B. J. (2018). Recent insights into targeting the IL-6 cytokine family in inflammatory diseases and cancer. Nat. Rev. Immunol. 18, 773–789. 10.1038/s41577-018-0066-7, PMID: 30254251

[ref44] Kakhki R. K. Kakhki M. K. Neshani A. (2020). COVID-19 target: A specific target for novel coronavirus detection. Gene Rep. 20:100740. 10.1016/j.genrep.2020.100740, PMID: 32510005 PMC7261075

[ref45] Kanai R. Zaupa C. Sgubin D. Antoszczyk S. J. Martuza R. L. Wakimoto H.. (2012). Effect of gamma34.5 deletions on oncolytic herpes simplex virus activity in brain tumors. J. Virol. 86, 4420–4431. 10.1128/JVI.00017-12, PMID: 22345479 PMC3318611

[ref46] Kang R. Zeh H. J. Lotze M. T. Tang D. (2011). The Beclin 1 network regulates autophagy and apoptosis. Cell Death Differ. 18, 571–580. 10.1038/cdd.2010.191, PMID: 21311563 PMC3131912

[ref47] Kim J. M. Chung Y. S. Jo H. J. Lee N. J. Kim M. S. Woo S. H.. (2020). Identification of coronavirus isolated from a patient in Korea with COVID-19. Osong. Public Health Res. Perspect. 11, 3–7. 10.24171/j.phrp.2020.11.1.02, PMID: 32149036 PMC7045880

[ref48] Kim J. H. Kim T. H. Lee H. C. Nikapitiya C. Uddin M. B. Park M. E.. (2017). Rubicon modulates antiviral type I interferon (IFN) signaling by targeting IFN regulatory factor 3 dimerization. J. Virol. 91, e00248–e00317. 10.1128/JVI.00248-17, PMID: 28468885 PMC5487567

[ref49] Kyei G. B. Dinkins C. Davis A. S. Roberts E. Singh S. B. Dong C.. (2009). Autophagy pathway intersects with HIV-1 biosynthesis and regulates viral yields in macrophages. J. Cell Biol. 186, 255–268. 10.1083/jcb.200903070, PMID: 19635843 PMC2717652

[ref50] Lake M. A. (2020). What we know so far: COVID-19 current clinical knowledge and research. Clin. Med. 20, 124–127. 10.7861/clinmed.2019-coron, PMID: 32139372 PMC7081812

[ref51] Law H. K. Cheung C. Y. Ng H. Y. Sia S. F. Chan Y. O. Luk W.. (2005a). Chemokine up-regulation in SARS-coronavirus-infected, monocyte-derived human dendritic cells. Blood 106, 2366–2374. 10.1182/blood-2004-10-4166, PMID: 15860669 PMC1895271

[ref52] Law P. T. W. Wong C. H. Au T. C. C. Chuck C. P. Kong S. K. Chan P. K. S.. (2005b). The 3a protein of severe acute respiratory syndrome-associated coronavirus induces apoptosis in Vero E6 cells. J. Gen. Virol. 86, 1921–1930. 10.1099/vir.0.80813-0, PMID: 15958670

[ref53] Leiva-Juarez M. M. Kolls J. K. Evans S. E. (2018). Lung epithelial cells: therapeutically inducible effectors of antimicrobial defense. Mucosal Immunol. 11, 21–34. 10.1038/mi.2017.71, PMID: 28812547 PMC5738267

[ref54] Levine B. Kroemer G. (2019). Biological functions of autophagy genes: A disease perspective. Cell 176, 11–42. 10.1016/j.cell.2018.09.048, PMID: 30633901 PMC6347410

[ref55] Li X. Xu S. Yu M. Wang K. Tao Y. Zhou Y.. (2020). Risk factors for severity and mortality in adult COVID-19 inpatients in Wuhan. J. Allergy Clin. Immunol. 146, 110–118. 10.1016/j.jaci.2020.04.006, PMID: 32294485 PMC7152876

[ref56] Liang Q. Chang B. Brulois K. F. Castro K. Min C. K. Rodgers M. A.. (2013). Kaposi’s sarcoma-associated herpesvirus K7 modulates Rubicon-mediated inhibition of autophagosome maturation. J. Virol. 87, 12499–12503. 10.1128/JVI.01898-13, PMID: 24027317 PMC3807930

[ref57] Lim J. Jeon S. Shin H. Y. Kim M. J. Seong Y. M. Lee W. J.. (2020). Case of the index patient who caused tertiary transmission of COVID-19 infection in Korea: the application of Lopinavir/ritonavir for the treatment of COVID-19 infected pneumonia monitored by quantitative RT-PCR. J. Korean Med. Sci. 35:e79. 10.3346/jkms.2020.35.e79, PMID: 32056407 PMC7025910

[ref58] Lippi G. Lavie C. J. Sanchis-Gomar F. (2020). Cardiac troponin I in patients with coronavirus disease 2019 (COVID-19): evidence from a meta-analysis. Prog. Cardiovasc. Dis. 63, 390–391. 10.1016/j.pcad.2020.03.001, PMID: 32169400 PMC7127395

[ref59] Liu F. Xu A. Zhang Y. Xuan W. Yan T. Pan K.. (2020). Patients of COVID-19 may benefit from sustained Lopinavir-combined regimen and the increase of eosinophil may predict the outcome of COVID-19 progression. Int. J. Infect. Dis. 95, 183–191. 10.1016/j.ijid.2020.03.013, PMID: 32173576 PMC7193136

[ref60] Lu R. Zhao X. Li J. Niu P. Yang B. Wu H. (2020). Genomic characterisation and epidemiology of 2019 novel coronavirus: implications for virus origins and receptor binding. Lancet 395, 565–574. 10.1016/S0140-6736(20)30251-8 32007145 PMC7159086

[ref61] Mao L. Jin H. Wang M. Hu Y. Chen S. He Q.. (2020). Neurologic manifestations of hospitalized patients with coronavirus disease 2019 in Wuhan, China. JAMA Neurol. 77, 683–690. 10.1001/jamaneurol.2020.1127, PMID: 32275288 PMC7149362

[ref62] Matsuyama S. Ujike M. Morikawa S. Tashiro M. Taguchi F. (2005). Protease-mediated enhancement of severe acute respiratory syndrome coronavirus infection. Proc. Natl. Acad. Sci. U. S. A. 102, 12543–12547. 10.1073/pnas.0503203102, PMID: 16116101 PMC1194915

[ref63] Mauthe M. Orhon I. Rocchi C. Zhou X. Luhr M. Hijlkema K. J.. (2018). Chloroquine inhibits autophagic flux by decreasing autophagosome-lysosome fusion. Autophagy 14, 1435–1455. 10.1080/15548627.2018.1474314, PMID: 29940786 PMC6103682

[ref64] Melody M. Nelson J. Hastings J. Propst J. Smerina M. Mendez J.. (2020). Case report: use of lenzilumab and tocilizumab for the treatment of coronavirus disease 2019. Immunotherapy 12, 1121–1126. 10.2217/imt-2020-0136, PMID: 32546029 PMC7319491

[ref65] Meo S. A. Klonoff D. C. Akram J. (2020). Efficacy of Chloroquine and hydroxychloroquine in the treatment of COVID-19. Eur. Rev. Med. Pharmacol. Sci. 24, 4539–4547. 10.26355/eurrev_202004_21038, PMID: 32373993

[ref66] Millet J. K. Whittaker G. R. (2015). Host cell proteases: critical determinants of coronavirus tropism and pathogenesis. Virus Res. 202, 120–134. 10.1016/j.virusres.2014.11.021, PMID: 25445340 PMC4465284

[ref67] Molina J. M. Delaugerre C. Le Goff J. Mela-Lima B. Ponscarme D. Goldwirt L.. (2020). No evidence of rapid antiviral clearance or clinical benefit with the combination of hydroxychloroquine and azithromycin in patients with severe COVID-19 infection. Med. Mal. Infect. 50:384. 10.1016/j.medmal.2020.03.006, PMID: 32240719 PMC7195369

[ref68] Musarrat F. Chouljenko V. Dahal A. Nabi R. Chouljenko T. Jois S. D.. (2020). The anti-HIV drug nelfinavir mesylate (Viracept) is a potent inhibitor of cell fusion caused by the SARSCoV-2 spike (S) glycoprotein warranting further evaluation as an antiviral against COVID-19 infections. J. Med. Virol. 10.1002/jmv.25985, PMID: [Epub ahead of print] 32374457 PMC7267418

[ref69] Nascimbeni A. C. Codogno P. Morel E. (2017). Local detection of PtdIns3P at autophagosome biogenesis membrane platforms. Autophagy 13, 1602–1612. 10.1080/15548627.2017.1341465, PMID: 28813193 PMC5612047

[ref70] Nelson C. A. Pekosz A. Lee C. A. Diamond M. S. Fremont D. H. (2005). Structure and intracellular targeting of the SARS-coronavirus Orf7a accessory protein. Structure 13, 75–85. 10.1016/j.str.2004.10.010, PMID: 15642263 PMC7125549

[ref71] Neuman B. W. Adair B. D. Yoshioka C. Quispe J. D. Orca G. Kuhn P.. (2006). Supramolecular architecture of severe acute respiratory syndrome coronavirus revealed by electron cryomicroscopy. J. Virol. 80, 7918–7928. 10.1128/JVI.00645-06, PMID: 16873249 PMC1563832

[ref72] Nutho B. Mahalapbutr P. Hengphasatporn K. Pattaranggoon N. C. Simanon N. Shigeta Y.. (2020). Why are Lopinavir and ritonavir effective against the newly emerged coronavirus 2019? Atomistic insights into the inhibitory mechanisms. Biochemistry 59, 1769–1779. 10.1021/acs.biochem.0c00160, PMID: 32293875

[ref73] Pan L. Mu M. Yang P. Sun Y. Wang R. Yan J.. (2020). Clinical characteristics of COVID-19 patients with digestive symptoms in Hubei, China: A descriptive, cross-sectional, multicenter study. Am. J. Gastroenterol. 115, 766–773. 10.14309/ajg.0000000000000620, PMID: 32287140 PMC7172492

[ref74] Parang K. El-Sayed N. S. Kazeminy A. J. Tiwari R. K. (2020). Comparative antiviral activity of Remdesivir and anti-HIV nucleoside analogs against human coronavirus 229E (HCoV-229E). Molecules 25:2343. 10.3390/molecules25102343, PMID: 32429580 PMC7287735

[ref75] Reggiori F. Monastyrska I. Verheije M. H. Cali T. Ulasli M. Bianchi S.. (2010). Coronaviruses hijack the LC3-I-positive EDEMosomes, ER-derived vesicles exporting short-lived ERAD regulators, for replication. Cell Host Microbe 7, 500–508. 10.1016/j.chom.2010.05.013, PMID: 20542253 PMC7103375

[ref76] Rockx B. Kuiken T. Herfst S. Bestebroer T. Lamers M. M. Oude Munnink B. B.. (2020). Comparative pathogenesis of COVID-19, MERS, and SARS in a nonhuman primate model. Science 368, 1012–1015. 10.1126/science.abb7314, PMID: 32303590 PMC7164679

[ref77] Rothlin R. P. Vetulli H. M. Duarte M. Pelorosso F. G. (2020). Telmisartan as tentative angiotensin receptor blocker therapeutic for COVID-19. Drug Dev. Res. 81, 768–770. 10.1002/ddr.21679, PMID: 32356926 PMC7267340

[ref78] Ruan Q. Yang K. Wang W. Jiang L. Song J. (2020a). Clinical predictors of mortality due to COVID-19 based on an analysis of data of 150 patients from Wuhan, China. Intensive Care Med. 46, 846–848. 10.1007/s00134-020-05991-x, PMID: 32125452 PMC7080116

[ref79] Ruan Q. Yang K. Wang W. Jiang L. Song J. (2020b). Correction to: clinical predictors of mortality due to COVID-19 based on an analysis of data of 150 patients from Wuhan, China. Intensive Care Med. 46, 1294–1297. 10.1007/s00134-020-06028-z, PMID: 32253449 PMC7131986

[ref80] Savarino A. Di Trani L. Donatelli I. Cauda R. Cassone A. (2006). New insights into the antiviral effects of chloroquine. Lancet Infect. Dis. 6, 67–69. 10.1016/S1473-3099(06)70361-9, PMID: 16439323 PMC7129107

[ref81] Schoeman D. Fielding B. C. (2019). Coronavirus envelope protein: current knowledge. Virol. J. 16:69. 10.1186/s12985-019-1182-0, PMID: 31133031 PMC6537279

[ref82] Sciascia S. Apra F. Baffa A. Baldovino S. Boaro D. Boero R. (2020). Pilot prospective open, single-arm multicentre study on off-label use of tocilizumab in patients with severe COVID-19. Clin. Exp. Rheumatol. 38, 529–532. PMID: 32359035

[ref83] Shang J. Ye G. Shi K. Wan Y. Luo C. Aihara H.. (2020). Structural basis of receptor recognition by SARS-CoV-2. Nature 581, 221–224. 10.1038/s41586-020-2179-y, PMID: 32225175 PMC7328981

[ref84] Sheahan T. P. Sims A. C. Graham R. L. Menachery V. D. Gralinski L. E. Case J. B.. (2017). Broad-spectrum antiviral GS-5734 inhibits both epidemic and zoonotic coronaviruses. Sci. Transl. Med. 9:eaal3653. 10.1126/scitranslmed.aal3653, PMID: 28659436 PMC5567817

[ref85] Sheahan T. P. Sims A. C. Leist S. R. Schafer A. Won J. Brown A. J. (2020). Comparative therapeutic efficacy of remdesivir and combination lopinavir, ritonavir, and interferon beta against MERS-CoV. Nat. Commun. 11:222. 10.1038/s41467-019-13940-6 31924756 PMC6954302

[ref86] Shi C. S. Nabar N. R. Huang N. N. Kehrl J. H. (2019). SARS-coronavirus open Reading frame-8b triggers intracellular stress pathways and activates NLRP3 inflammasomes. Cell Death Dis. 5:101. 10.1038/s41420-019-0181-7 PMC6549181 31231549

[ref87] Shi S. Qin M. Shen B. Cai Y. Liu T. Yang F.. (2020). Association of Cardiac Injury with Mortality in hospitalized patients with COVID-19 in Wuhan, China. JAMA Cardiol. 5, 802–810. 10.1001/jamacardio.2020.0950, PMID: 32211816 PMC7097841

[ref88] Singh A. K. Singh A. Singh R. Misra A. (2020). Hydroxychloroquine in patients with COVID-19: A systematic review and meta-analysis. Diabetes Metab. Syndr. 14, 589–596. 10.1016/j.dsx.2020.05.017, PMID: 32417708 PMC7215156

[ref89] Siu Y. L. Teoh K. T. Lo J. Chan C. M. Kien F. Escriou N.. (2008). The M, E, and N structural proteins of the severe acute respiratory syndrome coronavirus are required for efficient assembly, trafficking, and release of virus-like particles. J. Virol. 82, 11318–11330. 10.1128/JVI.01052-08, PMID: 18753196 PMC2573274

[ref90] Sun P. Qie S. Liu Z. Ren J. Li K. Xi J. (2020). Clinical characteristics of hospitalized patients with SARS-CoV-2 infection: A single arm meta-analysis. J. Med. Virol. 92, 612–617. 10.1002/jmv.25735, PMID: 32108351 PMC7228255

[ref91] Talloczy Z. Jiang W. Virgin H. W. T. Leib D. A. Scheuner D. Kaufman R. J.. (2002). Regulation of starvation- and virus-induced autophagy by the eIF2alpha kinase signaling pathway. Proc. Natl. Acad. Sci. U. S. A. 99, 190–195. 10.1073/pnas.012485299, PMID: 11756670 PMC117537

[ref92] Tan L. Wang Q. Zhang D. Ding J. Huang Q. Tang Y. Q.. (2020). Correction: Lymphopenia predicts disease severity of COVID-19: a descriptive and predictive study. Signal Transduct. Target. Ther. 5:61. 10.1038/s41392-020-0159-1, PMID: 32377400 PMC7189020

[ref93] Teijaro J. R. (2017). Cytokine storms in infectious diseases. Semin. Immunopathol. 39, 501–503. 10.1007/s00281-017-0640-2, PMID: 28674818 PMC7079934

[ref94] Temesgen Z. Assi M. Vergidis P. Rizza S. A. Bauer P. R. Pickering B. W. (2020). First Clinical Use of Lenzilumab to Neutralize GM-CSF in Patients with Severe COVID-19 Pneumonia. medRxiv [Preprint]. 10.1101/2020.06.08.20125369

[ref95] Tian Y. Rong L. Nian W. He Y. (2020). Review article: gastrointestinal features in COVID-19 and the possibility of faecal transmission. Aliment. Pharmacol. Ther. 51, 843–851. 10.1111/apt.15731, PMID: 32222988 PMC7161803

[ref96] Tomazini B. M. Maia I. S. Cavalcanti A. B. Berwanger O. Rosa R. G. Veiga V. C.. (2020). Effect of dexamethasone on days alive and ventilator-free in patients with moderate or severe acute respiratory distress syndrome and COVID-19: the CoDEX randomized clinical trial. JAMA 324, 1307–1316. 10.1001/jama.2020.17021, PMID: 32876695 PMC7489411

[ref97] Tran D. H. Sugamata R. Hirose T. Suzuki S. Noguchi Y. Sugawara A.. (2019). Azithromycin, a 15-membered macrolide antibiotic, inhibits influenza A(H1N1)pdm09 virus infection by interfering with virus internalization process. J. Antibiot. 72, 759–768. 10.1038/s41429-019-0204-x, PMID: 31300721

[ref98] Tsoi H. Li L. Chen Z. S. Lau K. F. Tsui S. K. Chan H. Y. (2014). The SARS-coronavirus membrane protein induces apoptosis via interfering with PDK1-PKB/Akt signalling. Biochem. J. 464, 439–447. 10.1042/BJ20131461, PMID: 25271362

[ref99] Vincent M. J. Bergeron E. Benjannet S. Erickson B. R. Rollin P. E. Ksiazek T. G.. (2005). Chloroquine is a potent inhibitor of SARS coronavirus infection and spread. Virol. J. 2:69. 10.1186/1743-422X-2-69, PMID: 16115318 PMC1232869

[ref100] Wang M. Cao R. Zhang L. Yang X. Liu J. Xu M.. (2020c). Remdesivir and chloroquine effectively inhibit the recently emerged novel coronavirus (2019-nCoV) in vitro. Cell Res. 30, 269–271. 10.1038/s41422-020-0282-0, PMID: 32020029 PMC7054408

[ref101] Wang D. Hu B. Hu C. Zhu F. Liu X. Zhang J.. (2020b). Clinical characteristics of 138 hospitalized patients with 2019 novel coronavirus-infected pneumonia in Wuhan, China. JAMA 323, 1061–1069. 10.1001/jama.2020.1585, PMID: 32031570 PMC7042881

[ref102] Wang C. Li W. Drabek D. Okba N. M. A. van Haperen R. Osterhaus A.. (2020a). A human monoclonal antibody blocking SARS-CoV-2 infection. Nat. Commun. 11:2251. 10.1038/s41467-020-16256-y, PMID: 32366817 PMC7198537

[ref103] Wang Y. Zhang D. Du G. Du R. Zhao J. Jin Y. (2020d). Remdesivir in adults with severe COVID-19: a randomised, double-blind, placebo-controlled, multicentre trial. Lancet 395, 1569–1578. 10.1016/S0140-6736(20)31022-9 32423584 PMC7190303

[ref104] Wu P. Duan F. Luo C. Liu Q. Qu X. Liang L.. (2020). Characteristics of ocular findings of patients with coronavirus disease 2019 (COVID-19) in Hubei Province, China. JAMA Ophthalmol. 138, 575–578. 10.1001/jamaophthalmol.2020.1291, PMID: 32232433 PMC7110919

[ref105] Wu K. Zhang Q. Wu X. Lu W. Tang H. Liang Z.. (2017). Chloroquine is a potent pulmonary vasodilator that attenuates hypoxia-induced pulmonary hypertension. Br. J. Pharmacol. 174, 4155–4172. 10.1111/bph.13990, PMID: 28849593 PMC5659991

[ref106] Xu X. Han M. Li T. Sun W. Wang D. Fu B.. (2020b). Effective treatment of severe COVID-19 patients with tocilizumab. Proc. Natl. Acad. Sci. U. S. A. 117, 10970–10975. 10.1073/pnas.2005615117, PMID: 32350134 PMC7245089

[ref107] Xu H. Zhong L. Deng J. Peng J. Dan H. Zeng X. (2020a). High expression of ACE2 receptor of 2019-nCoV on the epithelial cells of oral mucosa. Int. J. Oral Sci. 12:8. 10.1038/s41368-020-0074-x 32094336 PMC7039956

[ref108] Yang N. Shen H. M. (2020). Targeting the Endocytic pathway and autophagy process as a novel therapeutic strategy in COVID-19. Int. J. Biol. Sci. 16, 1724–1731. 10.7150/ijbs.45498, PMID: 32226290 PMC7098027

[ref109] Yang Y. Xiong Z. Zhang S. Yan Y. Nguyen J. Ng B.. (2005). Bcl-xL inhibits T-cell apoptosis induced by expression of SARS coronavirus E protein in the absence of growth factors. Biochem. J. 392, 135–143. 10.1042/BJ20050698, PMID: 16048439 PMC1317672

[ref110] Ye M. Fu D. Ren Y. Wang F. Wang D. Zhang F.. (2020a). Treatment with convalescent plasma for COVID-19 patients in Wuhan, China. J. Med. Virol. 10.1002/jmv.25882, PMID: [Epub ahead of print] 32293713 PMC7262027

[ref111] Ye X. T. Luo Y. L. Xia S. C. Sun Q. F. Ding J. G. Zhou Y.. (2020b). Clinical efficacy of lopinavir/ritonavir in the treatment of coronavirus disease 2019. Eur. Rev. Med. Pharmacol. Sci. 24, 3390–3396. 10.26355/eurrev_202003_20706, PMID: 32271456

[ref112] Ye Z. Wong C. K. Li P. Xie Y. (2008). A SARS-CoV protein, ORF-6, induces caspase-3 mediated, ER stress and JNK-dependent apoptosis. Biochim. Biophys. Acta 1780, 1383–1387. 10.1016/j.bbagen.2008.07.009, PMID: 18708124 PMC7115782

[ref113] Yu B. Li C. Chen P. Zhou N. Wang L. Li J.. (2020). Low dose of hydroxychloroquine reduces fatality of critically ill patients with COVID-19. Sci. China Life Sci. 63, 1515–1521. 10.1007/s11427-020-1732-2, PMID: 32418114 PMC7228868

[ref114] Yuan X. Shan Y. Yao Z. Li J. Zhao Z. Chen J. (2006). Mitochondrial location of severe acute respiratory syndrome coronavirus 3b protein. Mol. Cell 21, 186–191. PMID: 16682811

[ref115] Zeng J. H. Liu Y. X. Yuan J. Wang F. X. Wu W. B. Li J. X.. (2020). First case of COVID-19 complicated with fulminant myocarditis: a case report and insights. Infection 48, 773–777. 10.1007/s15010-020-01424-5, PMID: 32277408 PMC7146072

[ref116] Zhang C. Wu Z. Li J. W. Zhao H. Wang G. Q. (2020). The cytokine release syndrome (CRS) of severe COVID-19 and Interleukin-6 receptor (IL-6R) antagonist Tocilizumab may be the key to reduce the mortality. Int. J. Antimicrob. Agents 55:105954. 10.1016/j.ijantimicag.2020.105954, PMID: 32234467 PMC7118634

[ref117] Zhao Z. Thackray L. B. Miller B. C. Lynn T. M. Becker M. M. Ward E.. (2007). Coronavirus replication does not require the autophagy gene ATG5. Autophagy 3, 581–585. 10.4161/auto.4782, PMID: 17700057

[ref118] Zheng Y. Y. Ma Y. T. Zhang J. Y. Xie X. (2020). COVID-19 and the cardiovascular system. Nat. Rev. Cardiol. 17, 259–260. 10.1038/s41569-020-0360-5, PMID: 32139904 PMC7095524

